# Efficacy and Safety of Alfuzosin as Medical Expulsive Therapy for Ureteral Stones: A Systematic Review and Meta-Analysis

**DOI:** 10.1371/journal.pone.0134589

**Published:** 2015-08-05

**Authors:** Chenli Liu, Guohua Zeng, Ran Kang, Wenqi Wu, Jiasheng Li, Kang Chen, Show P. Wan

**Affiliations:** Department of Urology, Minimally Invasive Surgery Center, The First Affiliated Hospital of Guangzhou Medical University, Guangdong Key Laboratory of Urology, Guangdong, China; Cardiff University, UNITED KINGDOM

## Abstract

**Background:**

Alfuzosin has been widely used to treat benign prostatic hyperplasia and prostatitis, and is claimed to be a selective agent for the lower urinary tract with low incidence of adverse side-effects and hypotensive changes. Recently, several randomized controlled trials have reported using Alfuzosin as an expulsive therapy of ureteral stones. Tamsulosin, another alpha blocker, has also been used as an agent for the expulsive therapy for ureteral stones. It is unclear whether alfuzosin has similar efficacy as Tamsulosin in the management of ureteral stones.

**Objective:**

To perform a systematic review and analysis of literatures comparing Alfuzosin with Tamsulosin or standard conservative therapy for the treatment of ureteral stones less than 10 mm in diameter.

**Methods:**

A systematic literature review was performed in December 2014 using Pubmed, Embase, and the Cochrane library databases to identify relevant studies. All randomized and controlled trials were included. A subgroup analysis was performed comparing Alfuzosin with control therapy on the management of distal ureteral stones.

**Results:**

Alfuzosin provided a significantly higher stone-free rate than the control treatments (RR: 1.85; 95% confidence interval [CI], 1.35–2.55; p<0.001), and a shorter stone expulsion time (Weighted mean difference [WMD]: -4.20 d, 95%CI, -6.19 to -2.21; p<0.001), but it has a higher complication rate (RR: 2.02; 95% CI, 1.30–3.15; p<0.01). When Alfuzosin was compared to Tamsulosin, there was no significant difference in terms of stone-free rate (RR: 0.90; 95% CI, 0.79–1.02; p = 0.09) as well as the stone expulsion time (WMD: 0.52 d, 95%CI, -1.61 to 2.64; p = 0.63). The adverse effects of Alfuzosin were similar to those of Tamsulosin (RR: 0.88; 95% CI, 0.61–1.26; p = 0.47).

**Conclusions:**

Alfuzosin is a safe and effective agent for the expulsive therapy of ureteral stones smaller than 10 mm in size. It is more effective than therapeutic regiment without alpha blocker. It is equivalent to Tamsulosin in its effectiveness and safety profile. Adverse effects should always be kept in mind when use this class of drugs.

## Introduction

Urolithiasis is now one of the main public health problems world-wide. The prevalence is estimated to be 13% in the United States, 5–9% in Europe and 1–5% in Asia[1]. The total health care-related expenditure for individuals with a diagnosis of urolithiasis in the USA is increasing annually and with an estimated cost of $2.1 billion in the year 2000[2]. Most urinary stone related visits to the emergency department are for ureteral stones less than 5mm in diameter, majority of them can pass spontaneously with conservative treatment. For stones between 5 mm to 10 mm, medical expulsive therapy (MET) has been recommended to facilitate stone passage according to the 2013 European Association of Urology guidelines. In addition to the expulsive therapy, the stone size and location also affect the probability of spontaneous passage and the expulsion time of the ureteral stones.

For medical expulsive therapy, Tamsulosin had been shown to be an effective medication for the expulsive therapy of ureteral stones. This claim was supported by several meta-analysis of randomized controlled trials (RCTs) [3,4,5,6]. Recently, Alfuzosin has also been used as an expulsive agent in the treatment of ureteral stones,it was claimed to be a uroselective agent with a low incidence of adverse side-effects and blood pressure changes [7]. However, due to the small sample of each published studies, the claim of superiority of Alfuzosin could not be substantiated. Therefore, we performed a systematic review of RCTs to investigate the effect of Alfuzosin as an expulsive medication on the spontaneous passage of ureteral calculi.

## Materials and Methods

### Literature search and article selection

A systematic review was performed in December 2014 using PubMed, Embase, and Cochrane library databases to identify relevant randomized and controlled trials. Searches were restricted to publications in English involving adult population. The date range of articles searched was from 1966 to December 2014. Searches were performed in each database using the search strategy: ((alfuzosin) OR uroxatral) AND ureteral stone. Additional searches were carried out from reference lists of publications in this field.

**Fig 1:**
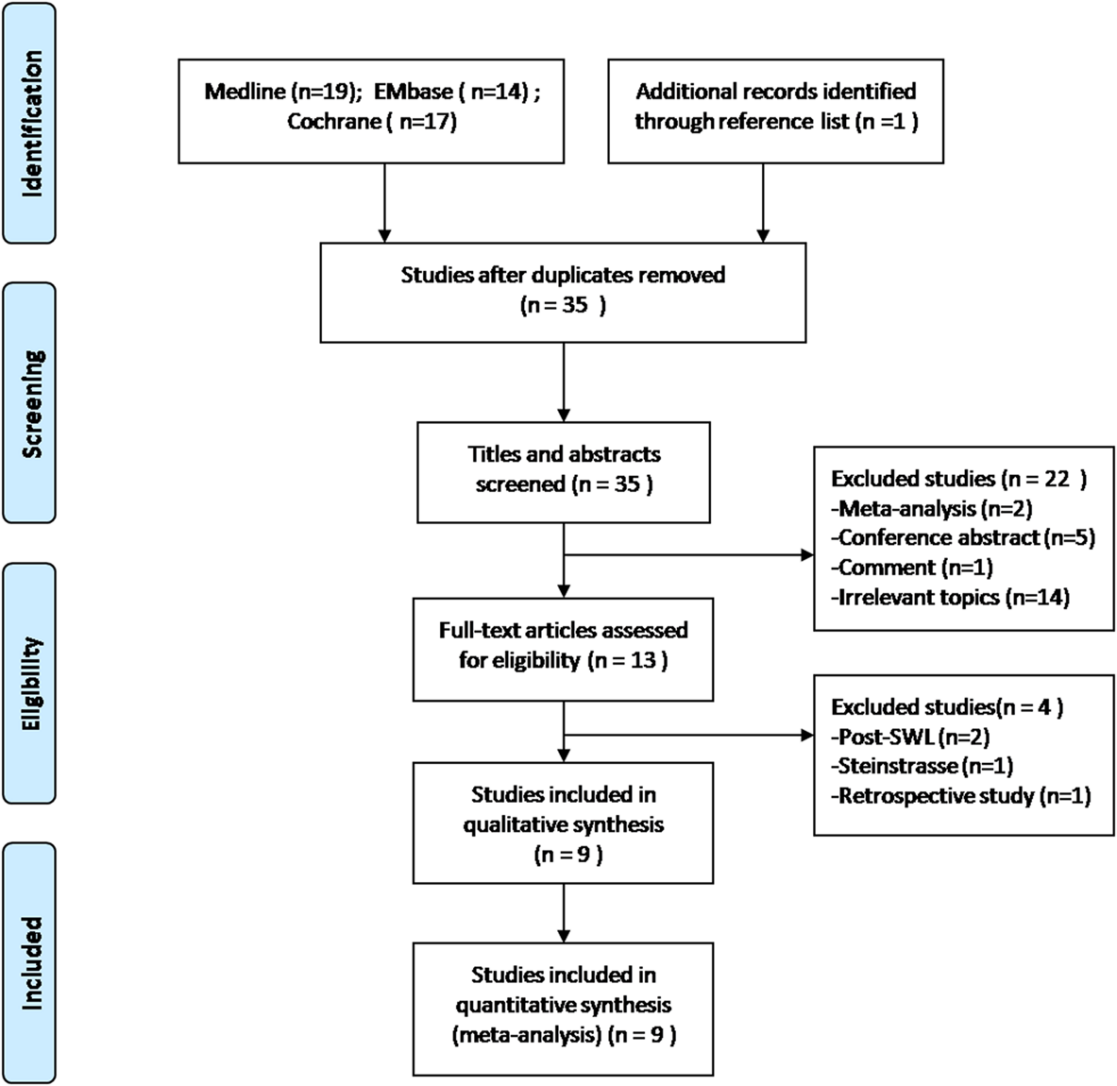
Flow diagram for reference search and selection of studies for analysis.

Article selection was carried out according to the search strategy recommended in the Preferred Reporting Items for Systematic Reviews and Meta-analysis criteria (www.prismastatement.org). Prospective randomized or controlled clinical trials comparing Alfuzosin and control conservative therapy to manage moderately sized ureteral calculi (<10mm) were included for further screening. Conference abstracts and studies reported on patients received shock wave lithotripsy (SWL) recently were excluded because they were not deemed to be methodologically appropriate. This review was performed by two independent reviewers; discrepancies during the process were resolved through discussion and consensus by the two reviewers. At the end, nine studies were entered in the analysis based on these criteria (Fig 1).

### Quality assessment and data extraction

The full text of the included studies was reviewed and assessed by two independent reviewers. The methodological quality of the trials was assessed using the Cochrane Collaboration criteria (Jadad scale) for RCTs[8]. Grading of Recommendations Assessment, Development and Evaluation (GRADE) system was used for rating quality of evidence[9]. Intervention related outcomes between the two treatments were compared.

A standardized data collection form was used for recording the extracted data. All pertinent information such as the period and location of the study performed, numbers of participants, and type and dose of the drugs administered, were recorded. Extracted data for the analysis included stone-free rate, stone expulsion time, need for analgesics, pain episodes, and complication rate. Stone expulsion rate, defined as the rate of patient with spontaneous stone expulsion without intervention during the study period, was considered as the primary outcome in this meta-analysis. The stone expulsion time and other indicators were considered as the secondary outcome measures.

### Statistical analysis

A meta-analysis was performed to assess the overall outcome of Alfuzosin therapy compared with control therapy. In addition, four of the nine studies also compared of Alfuzosin to Tamsulosin treatment, and the overall analysis of the two alpha-1blocking agents was performed independently. A subgroup analysis was performed comparing Alfuzosin with the control therapy on the management of distal ureteric stones. Risk ratios (RR) were used for binary variables; weighted mean difference (WMD) was used for the continuous data and displayed as means and standard deviations. The fixed effect model (Mantel-Haenszel method) was used for the pooled estimates, if no significant heterogeneity was detected; otherwise, the random effect model was used. The overall effects were determined by the z test, and p < 0.05 was considered statistically significant. The statistical heterogeneity across studies was assessed by the chi-square test and inconsistency (I^2^), and I^2^ >50% suggesting substantial statistical heterogeneity[10]. Review Manager software (RevMan.5.2, Cochrane Collaboration, Oxford, UK) was used for the data analysis.

## Results

### Study characteristics

Nine studies that met our selection criteria were entered for the analysis. There included 291 patients who received Alfuzosin 10 mg p.o, 134 cases received Tamsulosin 0.4mg p.o, and 280 cases received conservative therapy, Table 1. For stone passage, Alfuzosin was compared with control therapy in all nine studies. Four studies[11,12,13,14] compared the effects of Alfuzosin with those of Tamsulosin. All studies except one[15] stated that the mean stone size was greater than 4mm in both groups. There were no significant difference in terms of mean stone size between the Alfuzosin-treated patients and the control study patients (5.94 vs. 5.94mm, respectively) as well as between the Alfuzosin-treated and Tamsulosin-treated groups (5.98 vs. 5.61mm, respectively). The methodological quality of the included studies was quite high for six of the RTCs (Jadad scale: 4–5 of 5 points)[11,12,13,15,16,17] and medium quality for three (Jadad scale: 2–3 of 5 points)[14,18,19]. The quality of evidence was considered to be moderate according to the GRADE system. Two studies[11,18] reported the stones located in either the upper or lower ureter; the other seven studies[12,13,14,15,16,17,19] restricted to stones in the distal ureter. All except one trial[15] reported the additional medication administered for pain relief. Treatment time varied from two to six weeks. SWL or ureteroscopy was employed for patients who failed to pass stones during the follow-up period.

**Table 1:** Summary of comparative studies meeting inclusion criteria for analysis.

Study	Country	Study period	Paints (n)	Stone size (mm)	Stone location	Additional medications	I/C^#^(mg/day)	F (weeks)	Study quality*
**Pedro[15]**	USA	2008	34 35	3.96 3.67	LU	NR	Alfuzosin 10 mg Control	4	5
**Cha[12]**	Korean	2012	36 30 34	5.81 5.73 5.59	LU	Trospium chloride (50 mg, tablet) every 8 hours	Alfuzosin 10 mg Tamsulosin 0.4mg Control	4	5
**Ibrahim[11]**	Iraq	2013	40 40 32	5.94 5.58 5.65	UU LU	Diclofenac potassium orally 50 mg and/or diclofenac sodium as an intramuscular injection of 75 mg on demand	Alfuzosin 10 mg Tamsulosin 0.4 mg Control	4	4
**Gurbuz[16]**	Turkey	2011	34 33	6.83 7.13	LU	Drink at least 3 L of fluids daily; Diclofenac injection (75 mg) intramuscularly on demand for pain relief	Alfuzosin 10 mg Control	2	4
**Chau[18]**	China	2011	33 34	6.7 6.9	UU LU	Dologesic (paracetamol+dextropropoxyphene) 4 tablets daily on a demand basis for 2 weeks	Alfuzosin10 mg Control	6	3
**Agrawal[13]**	India	2008	34 34 34	6.70 6.17 6.35	LU	Drink at least 3 L of fluids daily; Diclofenac injection (75 mg) intramuscularly on demand for pain relief	Alfuzosin10 mg Tamsulosin 0.4 mg Control	4	5
**Ahmed[14]**	KSA	2010	30 30 28	5.47 4.97 5.39	LU	Diclofenac sodium (50 mg, tablet) every 12 hours for 1 week; Diclofenac sodium injection (75 mg, amp) as needed	Alfuzosin 10 mg Tamsulosin 0.4 mg Control	4	3
**Sameer[17]**	India	2014	35 35	6.26 6.37	LU	Diclofenac sodium (50 mg, tablet) every 12 hours for 1 week; Diclofenac sodium injection (75 mg, amp) as needed	Alfuzosin 10 mg Control	4	5
**Morua[19]**	Mexico	2009	15 15	5.80 6.43	LU	Buscopan 10 mg/8h plus ketorolac 10 mg/8h	Alfuzosin 10 mg Control	4	2

NR = not record; UU = upper ureter; LU = lower ureter; F = follow-up

^#^ Control group receiving placebo or dologesic

* Using Jadad scale (score from 0 to 5)

Diclofenac injections and oral non-steroidal anti-inflammatory drugs (NSAIDs) were widely used alone or in combination. Pain episodes and the need of analgesic were recorded as part of data collection in almost all of the studies. They were presented as averages with no standard deviations calculation. Seven of the nine studies showed no significant difference between the Alfuzosin and the control therapy in term of pain episodes and analgesics use (p>0.05, respectively) while as two studies revealed otherwise (p = 0.03, p<0.05, respectively). The difference between the Alfuzosin and Tamsulosin group was not statistically significant in terms of pain episodes in each study (p>0.05, respectively), Table 2.

**Table 2:** Analgaesic use and pain episodes in trails during the follow-up.

Studies		No. of Analgaesic Use (n)				No. of Pain Episodes(n)			
	Additional treatments	A	T	C	*p*	A	T	C	*p*
**Pedro[15]**	Opioidmedications (tablets)	8.63	-	9.41	0.45	-	-	-	
	Morphine equivalents	7.59	-	8.36	0.83				
**Cha[12]**	No record	-	-	-	-	-	-	-	
**Ibrahim[11]**	Diclofenac injections 75mg /Diclofenac sodium (tablets)	-	-	-	-	1.64	1.38	2.45	> 0.05*
**Gurbuz[16]**	Diclofenac(tablets)	4.36	-	3.75	0.57	-	-	-	
**Chau[18]**	Diclofenac injections75mg	17.0	-	16.0	0.87	-	-	-	
	NSAIDs (tablets)	1.5	-	6.9	0.03				
**Agrawal[13]**	Diclofenac injections75mg	1	0.88	6.2	<0.05*	0.82	0.58	5.5	<0.05*
**Ahmed[14]**	Diclofenac injection75mg	-	-	-	-	1.43	1.24	1.75	0.04
**Sameer[17]**	Diclofenac injection75mg	-	-	-	-	1.80	-	2.82	<0.001
**Morua[19]**	No record	-	-	-	-	-	-	-	

*Alfuzosin compared with control therapy A = Alfuzosin; T = Tamsulosin; C = control; NSAIDs = nonsteroidal antiinflammatory drugs

### Data synthesis

#### Overall analysis

**Fig 2:**
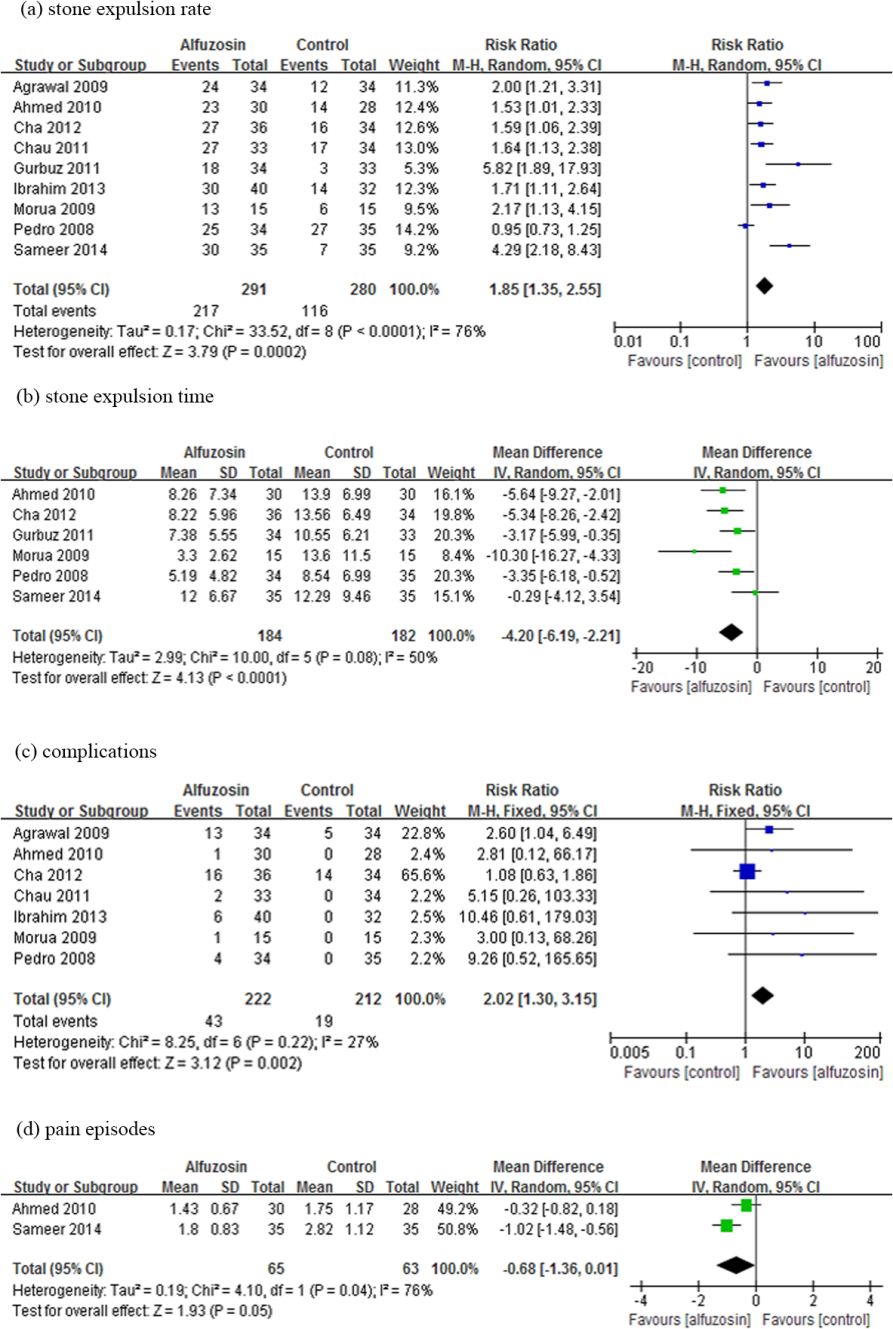
Forest plot of Alfuzosin versus control therapy. The central square of each horizontal line represents the RR or WMD for each study. The lines demonstrate the range of the 95% CI. The vertical line at an RR of 1 or at WMD of 0 is the line of no effect. % Weight indicates the influence exerted by each study on the pooled RR or WMD. CI = confidence interval; M-H = Mantel-Haenszel; IV = inverse variance; SD = standard deviation.

**Fig 3:**
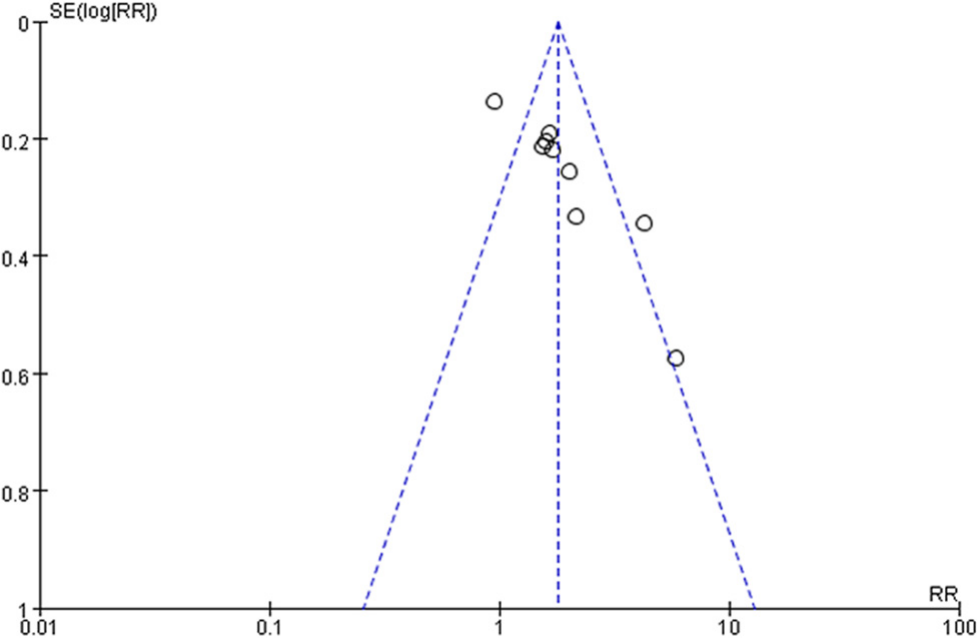
Funnel plot of Alfuzosin versus control therapy. Funnel plot for evaluation of publication bias. Vertical solid line represents the logarithmic transformation of the overall estimated treatment effect (ie, log [RR]), diagonal dotted lines represent pseudo–95% confidence limits for estimated treatment effect, and the circles represent treatment effects of each of the 9 studies. In the absence of publication bias, graph should represent a funnel, with individual studies clustered around the overall estimated treatment effect symmetrically.

There was a significantly higher stone expulsion rate following treatment with Alfuzosin as compared with control therapy (RR: 1.85; 95%CI, 1.35, 2.55; p<0.001). Alfuzosin also provided a shorter stone expulsion time (WMD: -4.20 d, 95%CI, -6.19 to -2.21; p<0.001). The pooled effect of adverse events reported by seven studies showed a higher complication rate for Alfuzosin treatment (RR: 2.02; 95% CI, 1.30–3.15; p<0.01). However, there was no significant difference between the two treatments in terms of pain episodes (WMD: -0.68, 95%CI, -1.48 to 0.01; p = 0.05) (Fig 2). The chi-square statistic for heterogeneity was significant (33.52 with df 8, p<0.0001, and I^2^ = 76%). The funnel plot was asymmetrical (Fig 3).

**Fig 4:**
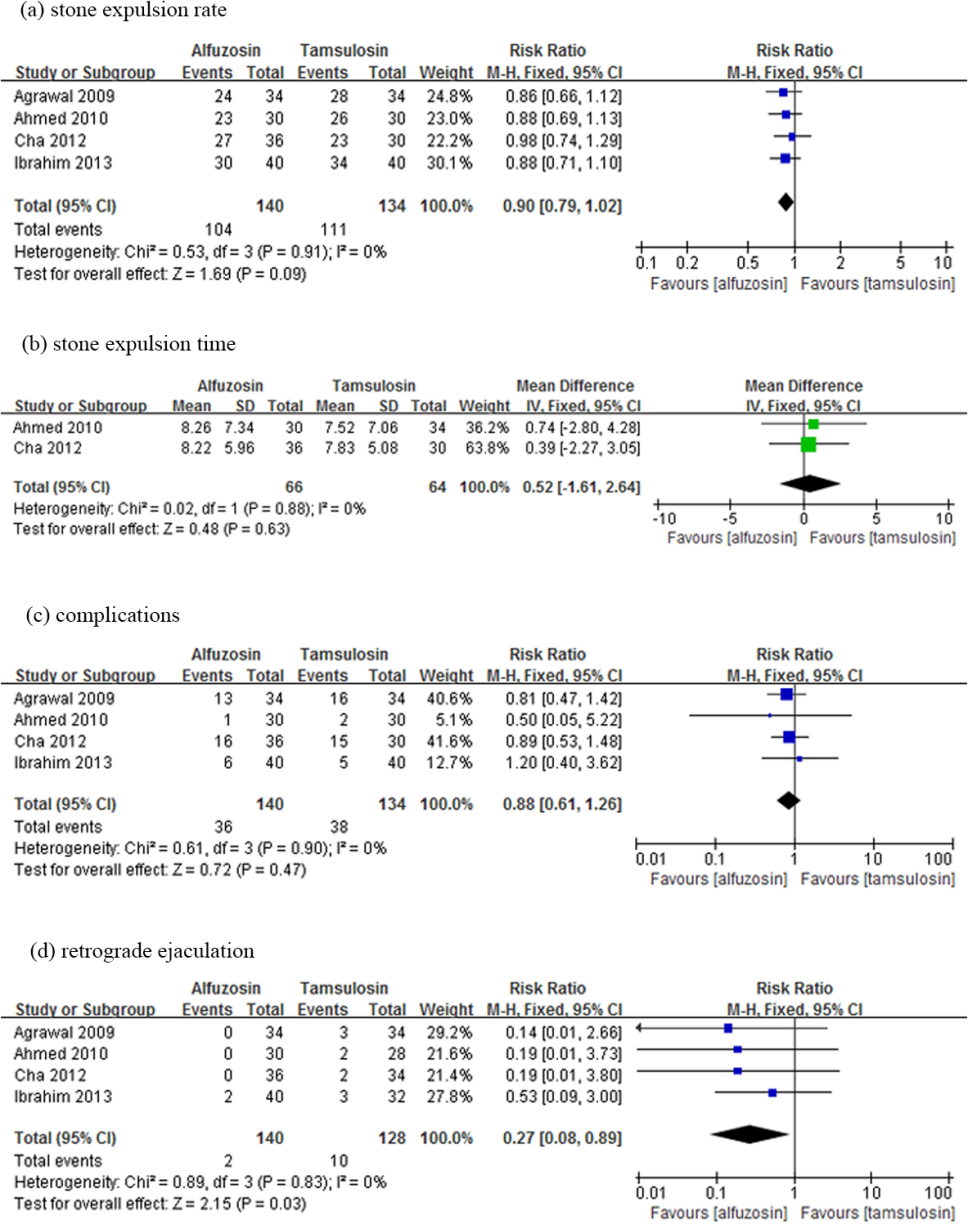
Forest plot of Alfuzosin versus Tamsulosin therapy. Refer to legend in Fig 2 for explanation.

There was no significant difference between Alfuzosin and Tamsulosin therapy in terms of stone expulsion rate (RR: 0.90; 95% CI, 0.79–1.02; p = 0.09) and stone expulsion time (WMD: 0.52 d, 95%CI, -1.61 to 2.64; p = 0.63). The difference in complication rates between Alfuzosin and Tamsulosin therapy was not statistically significant (RR: 0.88; 95% CI, 0.61–1.26; p = 0.47). Pooled analysis showed a lower frequency of retrograde ejaculation in the Alfuzosin-treated group than in the Tamsulosin-treated group (RR: 0.27; 95% CI, 0.08–0.89; p = 0.03) (Fig 4).

#### Subgroup and Sensitivity Analysis

**Fig 5:**
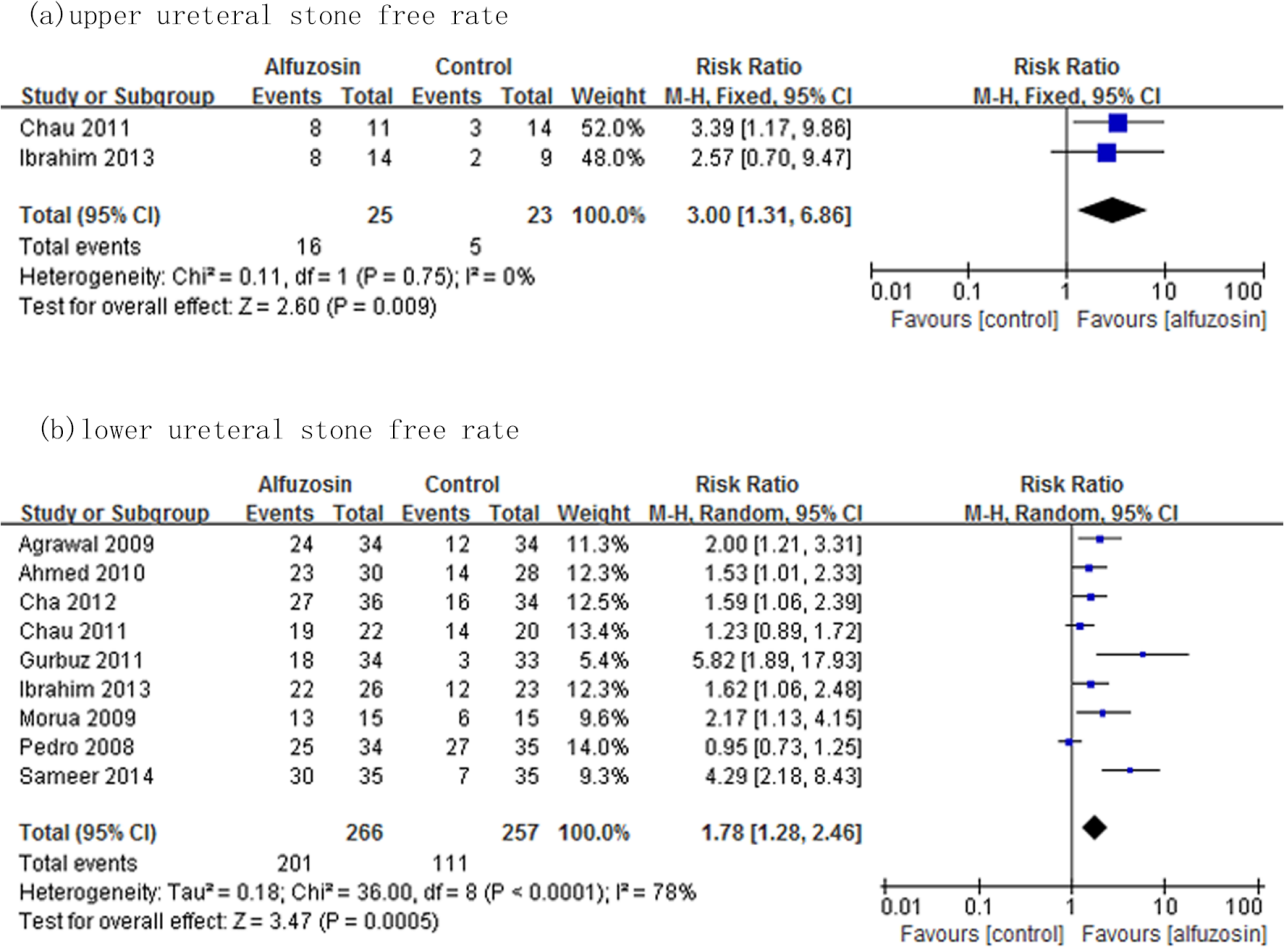
Forest plot of subgroup analysis categorized by stone locations. Refer to legend in Fig 2 for explanation.

Subgroup analysis categorized by stone locations (comparing the trials that included stones located in the upper ureter and lower ureter) showed that Alfuzosin provided a significantly higher stone expulsion rate than the control therapy for upper ureteral stones (RR: 3.00; 95%CI, 1.31–6.86; p = 0.004) as well as the lower ureteral stones (RR: 1.78; 95%CI, 1.28–2.46; p = 0.0005), respectively (Fig 5). Sensitivity analysis performed by sequential exclusion of each study from the analysis resulted in no significant changes in the pooled RR and/or in the precision of the effect estimates.

## Discussion

In this systematic review and meta-analysis, which included 9 studies with 705 patients, we noted that the use of Alfuzosin was associated with a significantly improved stone clearance rate and decreased the expulsion time. Furthermore the efficacy extended to stones located in both upper and lower ureter. Compared with patients who received control conservative treatment alone, patients who received Alfuzosin were 85% more likely to pass the ureteral stones. The stone expulsion time of Alfuzosin therapy for ureteric calculi was shorter in the pooled analysis, since the overall WMD of -4.20 days denoted a mean decrease in the expulsion time of 4.2 days.

To our knowledge, this is the first evidence-based analysis of RCTs to study the efficacy of Alfuzosin alone or in combination with other non-alpha blocker medication in the management of ureteral calculi. Alfuzosin exhibits no pharmacological uroselectivity for any of the alpha-1 subtype receptors, however, it did prove to have less side-effects and blood pressure changes as reported in the literature [7]. Thus, we focused on Alfuzosin treatment for promoting stone passage and attempted to find whether it has a similar effect in facilitating spontaneous stone expulsion with fewer side-effects than Tamsulosin. In the present pooled analysis of Alfuzosin and Tamsulosin treatment showed there was no statistically significant difference in terms of stone passage rate and expulsion time between the two drugs.

The sensitivity analysis of our study suggests that the effect estimate is robust. Potential sources of heterogeneity include the stone size and location. Subgroup analysis revealed a lower effect of heterogeneity when the study of mean stone size less than 4mm was excluded. In the subgroup analysis of stones by location, the effect of Alfuzosin was shown to be superior for upper ureteral stones than lower ureteral stones in respect to spontaneous stone passage rate (overall RR: 3.00 and overall RR: 1.78, respectively). It is opposite to that of Tamsulosin as a previous meta-analysis reported[6]. This might be attributed to that it is more difficult for upper ureter stones to spontaneous passage with conservative therapy. Our findings suggested Alfuzosin is an effective adjunctive therapy for ureteral stones regardless of stone size or location.

The present analysis based on the currently available RCTs revealed that Alfuzosin might significantly increase the adverse effects up to 102%, as compared with conservative therapy with an overall RR of 2.02, and 95% CI ranging from 1.30 to 3.15. The frequency of overall side-effects was not statistically different between Alfuzosin and Tamsulosin. Whereas retrograde ejaculation was approximately 70% lower in the Alfuzosin group than in the Tamsulosin group, this suggests Alfuzosin might have an advantage when used to treat male patients afflicting with ureteral stone.

The pain episodes and the need of analgesic are important indicators of the treatment effect for symptomatic relief. A significant decrease of pain episodes were reported by three RCTs[13,14,17] and lower analgesic requirement was reported in two studies[13,18]. However, in the pooled effect analysis there showed no statistically significant difference between Alfuzosin and conservative treatment. Therefore, a high quality evidence RCT is needed to verify this fact.

Publication bias is a potential limitation in any systematic review, and it might lead to overestimation of the therapy effect for the null or non-significant outcomes might not to be submitted and published [20]. Funnel plots in our analysis was asymmetric, this might be attributed to only the published studies included in this analysis. Alternatively, heterogeneity of treatment effects might be another plausible explanation [21]. Additionally, the quality of our data was moderate according to the GRADE system which decreases the evidence level of the therapeutic effect. Finally, the Jadad scores ranged from 2 to 5 among trials which reflects the poor overall quality of the studies reviewed.

## Conclusions

Alfuzosin is associated with significantly increased stone passage rate and decreased stone expulsion time. Its efficacy is comparable to that of Tamsulosin. In addition the effectiveness of the Alfuzosin therapy extends to stones in all locations. Adverse effects of Alfuzosin are similar to that of Tamsulosin with the exception of retrograde ejaculation. This suggests that Alfuzosin might have an advantage in treating male patients with ureteral stones. Nevertheless the adverse effects of Alfuzosin should always be kept in mind when prescribe this class of therapy. This study is a meta-analysis of published data. The best data should be derived from a high quality prospective random controlled trial. We are currently planning on such a project.

## Supporting Information

S1 PRISMA Checklist PRISMA 2009 Checklist. (DOC)

S1 File The search strategy used for literature search. (DOCX)
