# Supplementary material for: Efficacy and Safety of Alfuzosin as Medical Expulsive Therapy for Ureteral Stones: A Systematic Review and Meta-Analysis
Source: PLoS One. 2015 Aug 5;10(8):e0134589. doi: 10.1371/journal.pone.0134589 (PMC4526635; doi:10.1371/journal.pone.0134589)
Supplement: S1 File — (DOCX) [file pone.0134589.s002.docx]

Search strategy used in pubmed

#1 Search (alfuzosin) AND ("1966"[Date - Completion] : "2014"[Date - Completion]) 466

#2 Search (uroxatral) AND ("1966"[Date - Completion] : "2014"[Date - Completion]) 468

#3 Search (ureteral stone) AND ("1966"[Date - Completion] : "2014"[Date - Completion]) 8383

#4 Search ((((alfuzosin) AND ("1966"[Date - Completion] : "2014"[Date - Completion]))) OR ((uroxatral) AND ("1966"[Date - Completion] : "2014"[Date - Completion]))) AND ((ureteral stone) AND ("1966"[Date - Completion] : "2014"[Date - Completion])) 19

Search strategy used in Cochrane Library

#1 "alfuzosin":ti,ab,kw in Trials (Word variations have been searched) 157

#2 uroxatral in Trials 0

#3 ureteral stone in Trials 405

#4 (alfuzosin OR uroxatral) AND ureteral stone in Trials 17
